# Supplementary material for: Defective dystrophic thymus determines degenerative changes in skeletal muscle
Source: Nat Commun. 2021 Apr 8;12:2099. doi: 10.1038/s41467-021-22305-x (PMC8032677; doi:10.1038/s41467-021-22305-x)
Supplement: Supplementary file 1 — Supplementary Information [file 41467_2021_22305_MOESM1_ESM.pdf]

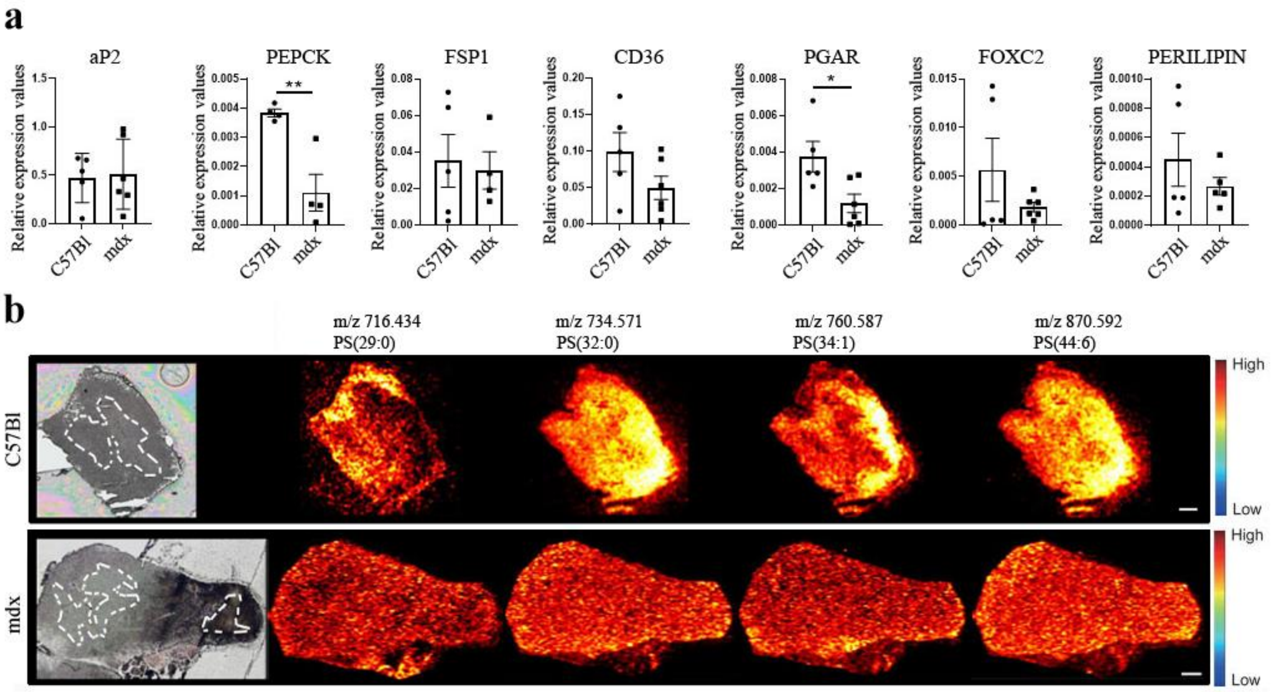

Supplementary Figure 1 – Adiposity is altered in mdx thymus

RT-qPCR experiments on thymus of mdx and C57Bl mice showed different expression of genes specifically involved in regulating thymic adiposity as *PGAR* and *PECPK* (**a**). Images captured with the iMScope TRIO described altered pattern of expression of different glycerophospholipids (as indicated by m/z values) between cortical and medullary zone (dashed white line) of mdx and C57Bl thymi (**b**).

Scale bar: 1 mm (**b**). The comparisons between the averages of the two groups were evaluated using two-sided Student's t-test. (**a**) * $p = 0.0221$, ** $p = 0.0055$. Data are presented as mean ± SD of three independent experiments with n = 5-6 (*foxc2*; *pgar*; *cd36*; *aP2*); n = 5 (*perilipin*); n = 4-5 (*fsp1*); n = 4 (*pecpk*) mice/group. Source data are provided as a Source Data file

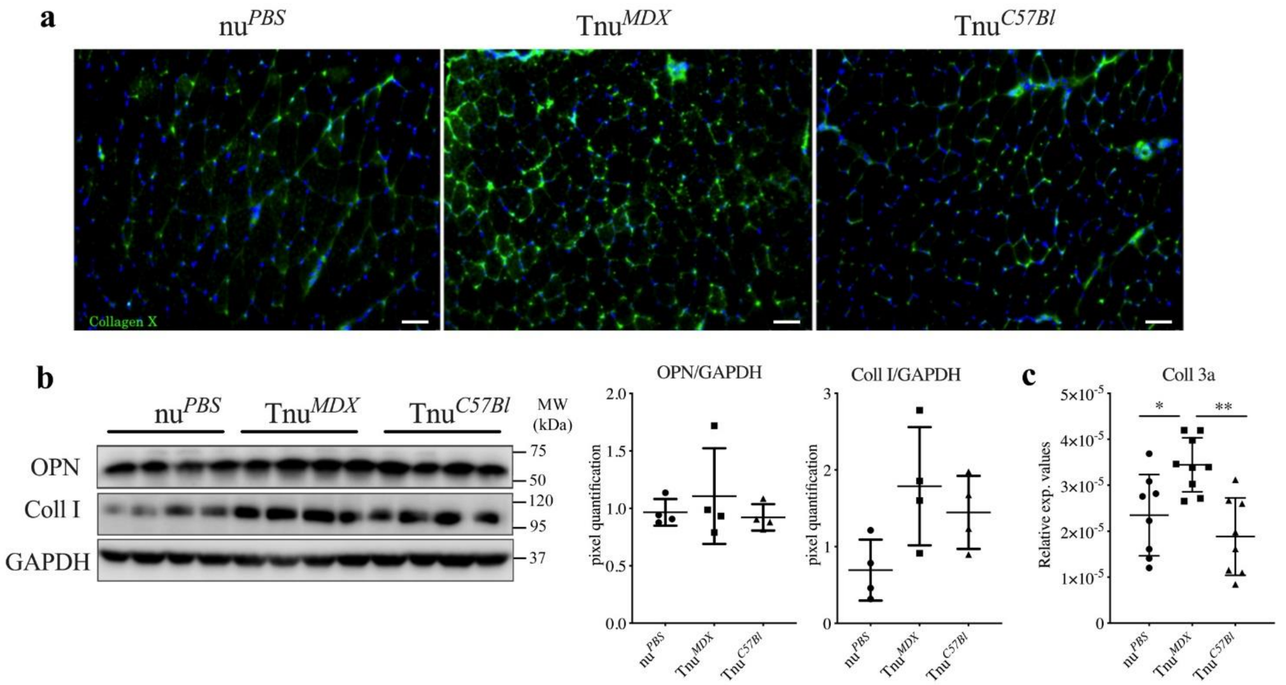

Supplementary Figure 2 – Muscle expression of collagen I and X as fibrotic markers of Tnu^{MDX} mice.

Representative immunostainings for collagen X of TA muscles of nu^{PBS}, Tnu^{MDX} and Tnu^{C57Bl} mice (**a**). Cropped image of representative western blot showing the expression of the collagen I, osteopontin (OPN) and GAPDH proteins in TAs of nu^{PBS}, Tnu^{MDX} and Tnu^{C57Bl} mice. Densitometric analyses are shown as collagen I/GAPDH ratio and OPN/GAPDH ratio (**b**). RT-qPCR experiments on muscle of thymus-transplanted nude mice revealed up-regulation of *collagen 3a* in Tnu^{mdx} (**c**).

Scale bar: 50µm (**a**). The comparisons between the averages of the two groups were evaluated using the One-way ANOVA. (**c**) * $p = 0.0211$, ** $p = 0.0012$. Data are presented as mean ± SD of three independent experiments with $n = 4$ (**b**), $n = 8\text{-}9$ (**c**) mice/group. Source data are provided as a Source Data file

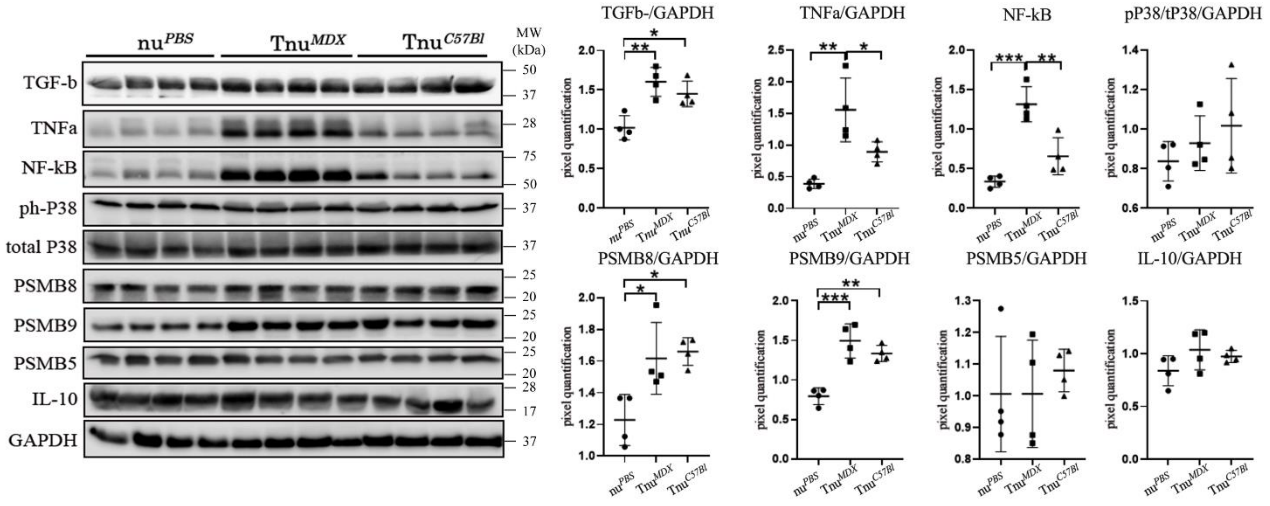

Supplementary Figure 3 - Inflammatory marker expression in muscles of Tnu^{MDX} mice.

Cropped image of representative WB showing the expression of the TNF-α, NF-kB, TGF-β, IL10, phospho-p38, p38, PSMB8, PSMB9, PSMB5 and GAPDH proteins in TAs of nu^{PBS}, Tnu^{MDX} and Tnu^{C57Bl} mice. Densitometric analyses shown that TNF-α and NF-kB were up-regulated in Tnu^{MDX} TA muscles while TGF-β did not change significantly.

The comparisons between the averages of the two groups were evaluated using the One-way ANOVA. PSMB8: * $p = 0.0239$. PSMB9: *** $p = 0.0003$. TGF-β: ** $p = 0.0020$; * $p = 0.0134$. TNF-α: ** $p = 0.0012$; * $p = 0.0335$. NF-kB: *** $p = 0.0001$; ** $p = 0.0023$. Data are presented as mean ± SD of three independent experiments with $n = 4$ mice/group. Source data are provided as a Source Data file

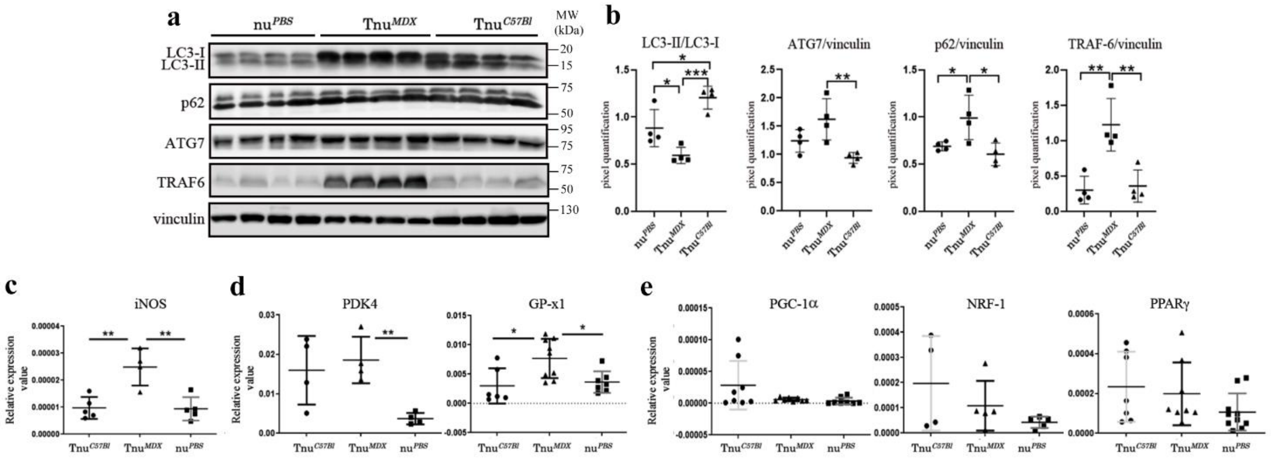

Supplementary Figure 4 - Oxidative stress and autophagy pathways are altered in muscles of Tnu^{MDX} mice

Cropped image of representative WB showed a dramatic decrease of the ratio of the autophagic protein LC3II/LC3I in the Tnu^{MDX} mice compared to nu^{PBS} and Tnu^{C57Bl} mice and, on contrary, an up-regulation of p62 and ATG-7 (**a, b**). Cropped image of representative western blot showed the over-expression of TRAF-6 in Tnu^{MDX} muscles (**a, b**). RT-qPCR showed the over-expression of *iNOS* in Tnu^{MDX} muscles (**c**). Metabolic muscle dysfunction in Tnu^{MDX} mice was demonstrated by the up-regulation of *pdk4* and *GPx1* genes (**d**). RT-qPCR experiments driven to investigate the mitochondrial biogenesis did not show any significant differences in the expression of *PGC-1*α, *PPAR*γ and *NRF-1* genes among treated mice (**e**).

The comparisons between the averages of the two groups were evaluated using the One-way ANOVA. (**b**) LC3II/LC3I: * $p = 0.0438$, *** $p = 0.0005$. ATG-7: * $p = 0.0090$. p62: * $p = 0.0123$ Tnu^{MDX} mice vs Tnu^{C57Bl} and * $p = 0.0420$ Tnu^{MDX} mice vs nu^{PBS}. TRAF-6: ** $p = 0.0041$ Tnu^{MDX} mice vs Tnu^{C57Bl} and ** $p = 0.0027$ Tnu^{MDX} mice vs nu^{PBS}. (**c**) ** $p = 0.0043$. (**d**) ** $p = 0.0027$; * $p = 0.0161$ Tnu^{MDX} mice vs nu^{PBS} and * $p = 0.0126$ Tnu^{MDX} mice vs Tnu^{C57Bl}. Data are presented as mean ± SD of three independent experiments with $n = 3$ (**a**); $n = 4$-5 (**c**); $n = 4$ (*pdk*) and $n = 6$-9 (*GP-x1*) (**d**); $n = 8$ (*pgc1*α), $n = 4$-5 (*nrf1*) and $n = 7$-9 (*ppar*α) (**e**) mice/group. Source data are provided as a Source Data file

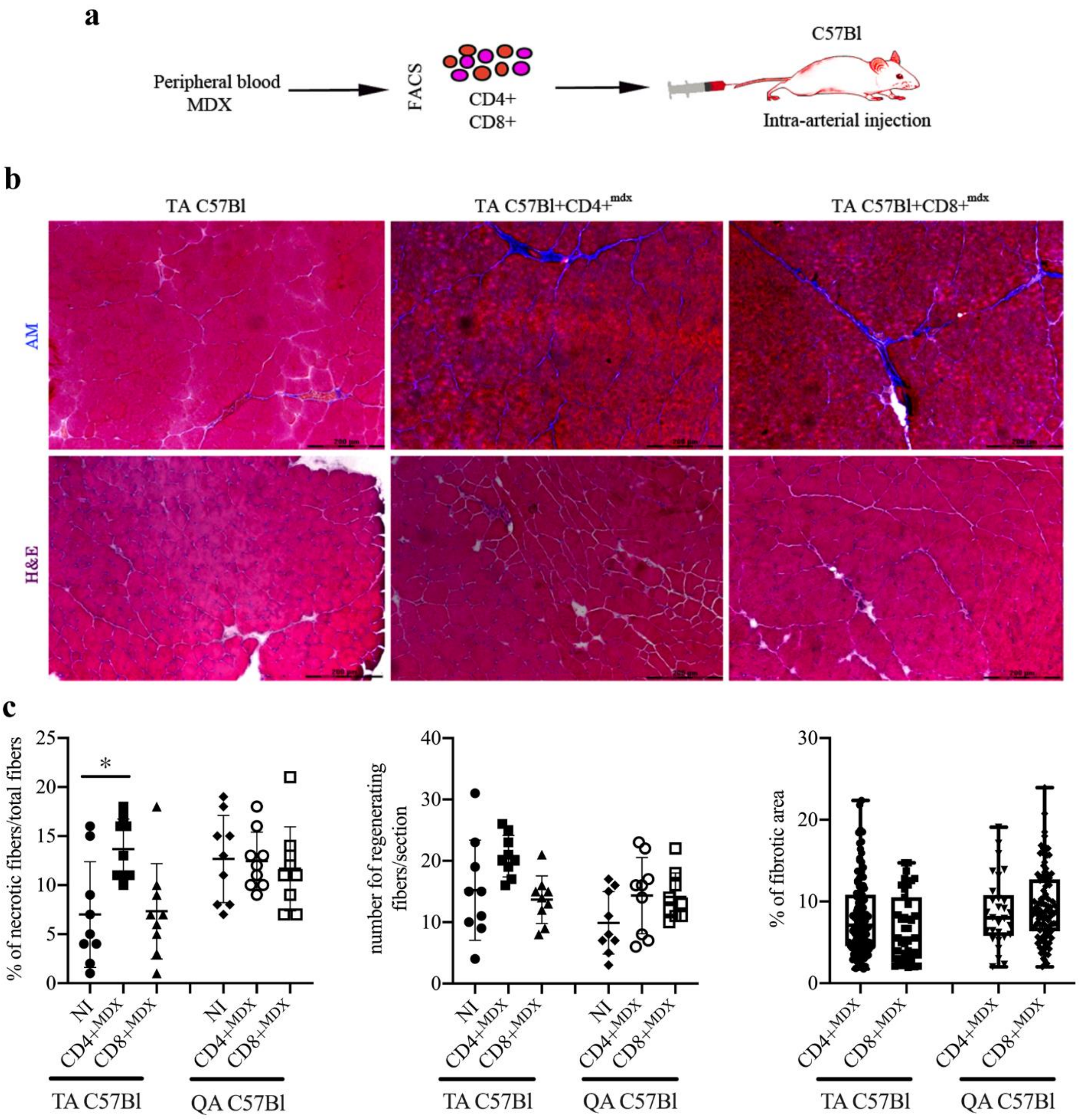

Supplementary Figure 5 – Intra-arterial delivery of dystrophic blood-derived T lymphocytes in healthy mice

Schematic overview of the experimental procedure (**a**). Representative H&E and AM staining of TA muscles of C57Bl mice intra-arterially injected with CD3+CD4+ (CD4+^{mdx}) and CD3+CD8+ (CD8+^{mdx}) T cells isolated from peripheral blood of mdx mice (**b**). Quantification of the necrotic myofibers, fibrotic areas and centrally-nucleated regenerating myofibers of the TA and QA muscles of untreated C57Bl (NI) and C57Bl intra-arterially injected with CD4+^{mdx} and CD8+^{mdx}. Boxes indicate 25th to 75th percentiles; whiskers indicate 5th to 95th percentiles; and the line indicates the median. For morphometric analysis, images were quantified with Image J software for each mouse (**c**).

Scale bar: 200μm (**b**). The comparisons between the averages of the two groups were evaluated using the One-way ANOVA. * p = 0.0257 (**c**). Data are presented as mean ± SD of three independent experiments with n = 3 mice/group with different slides counted/mice. Source data are provided as a Source Data file

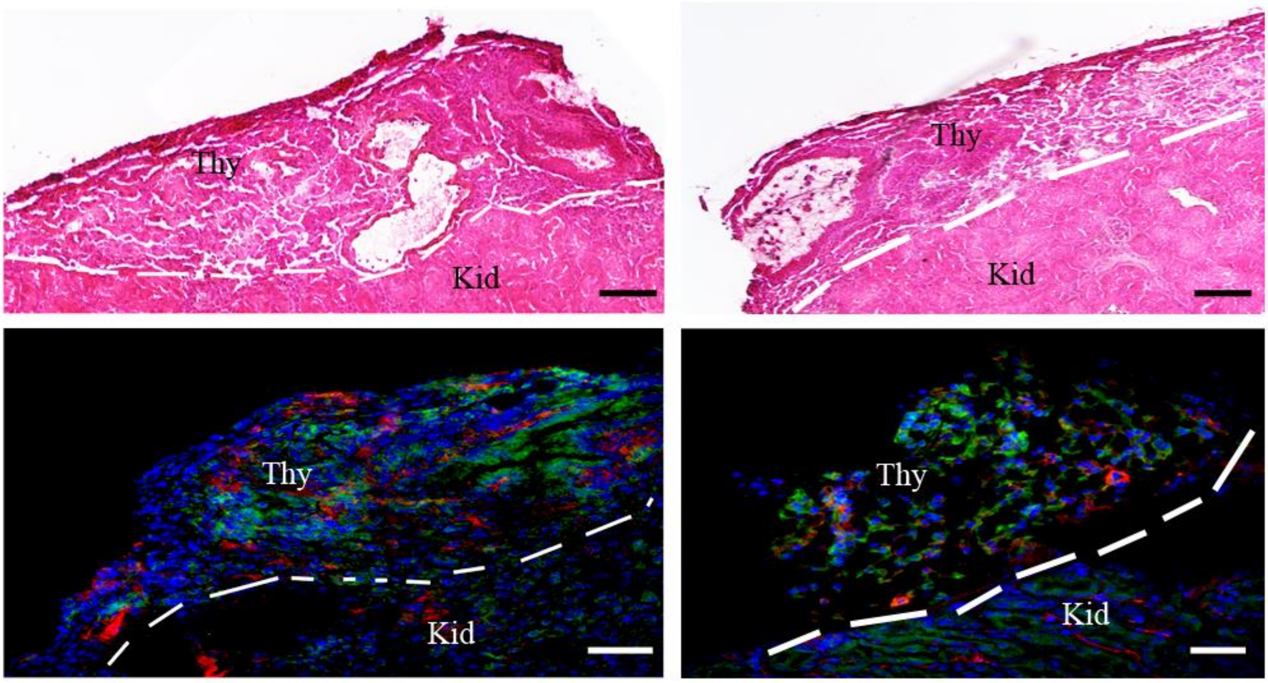

Supplementary Figure 6 – Characterization of kidneys transplanted with E17 thymi
Representative H&E staining of kidneys transplanted with E17 thymi from C57Bl (left panel) and mdx (right panel) mice, after 120 days. Grafted thymi (Thy) and kidneys (Kid) were confined in separated areas (dashed lines). Confocal microscope images showed the presence of CD3+ cells and TR7+ interstitial fibroblasts (red) within the grafted thymi (green). Scale bar: 20μm.

Supplementary Table 1 List of primers (5'→3') for RT-PCR

**mDys71-f** ACTCCTCCGCTCTAAGCGT
**mDys71-r** CTTCTGGAGCCCTTCTGAGC
**mDys116-f** ACACAGGAAGACATACCATG
**mDys116-r** TTCTGAACTGCTGGGAAATC
**mDys140-f** GAGGCATTGCTGACTGTTCT
**mDys140-r** TTAGCTGCTGCTCATCTCCA
**mDys260-f** GTGCAGCTACCTCTGTTCAG
**mDys260-r** CTGTGCAACATCAATTTGTGAA
**mDys427-f** GTTCATTGATGGAGACGGAA
**mDys427-r** GCACTTCAGCTTCTTCATCT

Supplementary Table 2 List of primers (5'→3') for RT-qPCR

**mRORγt-f** GACTGACAATCAGCAGGGATAA
**mRORγt-r** GGGAAATACAATGAGGTATTGAAAGG
**mp62-f** AGGCGCACTACCGCGAT
**mp62-r** CGTCACTGGAAAAGGCAACC
**mTbet-f** GATCATCACTAAGCAAGGAC
**mTbet-r** ACATCCACAAACATCCTGTA
**mMurF1-f** CAGAGGCAGTTGGATCGTCTATG
**mMurF1-r** TGAGGCAGAGTCTCTCTATGT
**mMYHCsl2-f** AAGCTGAGGAGGCTGAGGAAC
**mMYHCs12-r** TTCACCTGGGACTCAGCAATG
**matp2a1-f** TGTTTGTCCTATTTCGGGGTG
**matp2a1-r** AATCCGCACAAGCAGGTCTTC
**miNOS-f** CTCACTGGGACAGCACAGAA
**miNOS-r** GGCCTTGTGGTGAAGAGTGT
**mpdk4-f** GTCTCAATAGTGTCACCTGTGTAA
**mpdk4-r** CCTGGGCATTTAGCATCTATCT
**mGPx1-f** AGTTCGGACATCAGGAGAATGGCA
**mGPx1-r** TCACCATTCACCTCGCACTTCTCA
**mPGC1α-f** GCTAAACGACTCCGAGAACAA
**mPGC1α-r** ACTGACCCAAACATCATACCC
**mNRF1-f** GGCACTGTCTCACTTATCCAGGTT
**mNRF1-r** CAGCCACGGCAGAATAATTCA
**mPPARα-f** TGATTGGTTCCAGGCAATTAGA
**mPPARα-r** CACTCGTACAGTCAGTTCAGTC
**CoxVa-f** TTGATGCCTGGGAATTGCGTAAAG
**CoxVa-r** AACAACCTCCAAGATGCGAACAG
**CoxVIIb-f** TTTCAGGACGCTTTGCAAGG
**CoxVIIb-r** TGCTTCGAACTTGGAGACGG
**Col3a-f** CCTTAACATGTGTCTTTAAAGCCC
**Col3a-r** AAATGCTTTTAAAGGTGCTTCTCT
