# Supplementary material for: Defective dystrophic thymus determines degenerative changes in skeletal muscle
Source: Nat Commun. 2021 Apr 8;12:2099. doi: 10.1038/s41467-021-22305-x (PMC8032677; doi:10.1038/s41467-021-22305-x)
Supplement: Supplementary file 3 — Reporting summary [file 41467_2021_22305_MOESM3_ESM.pdf]

nature research

Corresponding author(s): Prof. Yvan Torrente

Last updated by author(s): Feb 1, 2021

Reporting Summary

Nature Research wishes to improve the reproducibility of the work that we publish. This form provides structure for consistency and transparency in reporting. For further information on Nature Research policies, see Authors & Referees and the Editorial Policy Checklist .

Statistics

For all statistical analyses, confirm that the following items are present in the figure legend, table legend, main text, or Methods section.

n/a	Confirmed	
☐	☒	The exact sample size (n) for each experimental group/condition, given as a discrete number and unit of measurement
☐	☒	A statement on whether measurements were taken from distinct samples or whether the same sample was measured repeatedly
☐	☒	The statistical test(s) used AND whether they are one- or two-sided<br>*Only common tests should be described solely by name; describe more complex techniques in the Methods section.*
☐	☒	A description of all covariates tested
☒	☐	A description of any assumptions or corrections, such as tests of normality and adjustment for multiple comparisons
☐	☒	A full description of the statistical parameters including central tendency (e.g. means) or other basic estimates (e.g. regression coefficient) AND variation (e.g. standard deviation) or associated estimates of uncertainty (e.g. confidence intervals)
☒	☐	For null hypothesis testing, the test statistic (e.g. F, t, r) with confidence intervals, effect sizes, degrees of freedom and P value noted<br>*Give P values as exact values whenever suitable.*
☐	☒	For Bayesian analysis, information on the choice of priors and Markov chain Monte Carlo settings
☒	☐	For hierarchical and complex designs, identification of the appropriate level for tests and full reporting of outcomes
☒	☐	Estimates of effect sizes (e.g. Cohen's d, Pearson's r), indicating how they were calculated

Our web collection on statistics for biologists contains articles on many of the points above.

Software and code

Policy information about availability of computer code

Data collection	Data collection was performed through ImageJ Software (NIH) version 1.46i
Data analysis	Data analysis was performed through ImageJ Software (NIH) version 1.46i

For manuscripts utilizing custom algorithms or software that are central to the research but not yet described in published literature, software must be made available to editors/reviewers. We strongly encourage code deposition in a community repository (e.g. GitHub). See the Nature Research guidelines for submitting code & software for further information.

Data

Policy information about availability of data

All manuscripts must include a data availability statement. This statement should provide the following information, where applicable:

- Accession codes, unique identifiers, or web links for publicly available datasets
- A list of figures that have associated raw data
- A description of any restrictions on data availability

The authors declare that the data supporting the findings of this study are available within the paper and its supplementary information files.

Field-specific reporting

Please select the one below that is the best fit for your research. If you are not sure, read the appropriate sections before making your selection.

☒ Life sciences ☐ Behavioural & social sciences ☐ Ecological, evolutionary & environmental sciences

For a reference copy of the document with all sections, see nature.com/documents/nr-reporting-summary-flat.pdf

Life sciences study design

All studies must disclose on these points even when the disclosure is negative.

Sample size	Sample size was selected to ensure an alpha value of 0.05
Data exclusions	No such exclusion in the paper
Replication	All the experiments in the paper were performed more than twice successfully except for some RT-qPCR replicates that were accordingly excluded from the analysis
Randomization	Randomization was used for animal studies since the animals were divided randomly by only one member of the group into cages and randomly destined to determined experiments
Blinding	Blinding test was performed for all the histological counts; the investigators were blinded to group allocation during data collection and/or analysis

Reporting for specific materials, systems and methods

We require information from authors about some types of materials, experimental systems and methods used in many studies. Here, indicate whether each material, system or method listed is relevant to your study. If you are not sure if a list item applies to your research, read the appropriate section before selecting a response.

Materials & experimental systems

n/a	Involved in the study	
☐	☒	Antibodies
☒	☐	Eukaryotic cell lines
☒	☐	Palaeontology
☐	☒	Animals and other organisms
☒	☐	Human research participants
☒	☐	Clinical data

Methods

n/a	Involved in the study	
☒	☐	ChIP-seq
☐	☒	Flow cytometry
☒	☐	MRI-based neuroimaging

Antibodies

Antibodies used

CD4-PE (Miltenyi Biotech-Germany)
CD3-APC (Miltenyi Biotech-Germany)
CD8-FITC (Miltenyi Biotech-Germany)
CD25-PerCP-Cy5.5 (BD Biosciences-Pharmingen, California, USA)
7AAD (BD Biosciences-Pharmingen, California, USA)
CD4 (Pacific Blue-A) (Biolegend, San Diego, CA, USA)
CD8 (APC-Cy7-A) (Biolegend, San Diego, CA, USA)
CD69 (Alexa Fluor 488-A) (Biolegend, San Diego, CA, USA)
TCRbeta (PE-A) (Biolegend, San Diego, CA, USA)
CD44 (Alexa Fluor 488-A) (Biolegend, San Diego, CA, USA)
Foxp3 (Alexa Fluor 488-A) (Biolegend, San Diego, CA, USA)
CD25 (APC-A) (Biolegend, San Diego, CA, USA)
CD45 (Pacific Blue, clone 30-F11) (Biolegend, San Diego, CA, USA)
EpCAM (PE-Cy7) (Biolegend, San Diego, CA, USA)
MHC-class II (PerCP/Cy5.5) (Biolegend, San Diego, CA, USA)
Ly51 (FITC) (Biolegend, San Diego, CA, USA)
UEA-1 fluorescein (Vector Laboratories, Burlingame, CA, USA)
Vinculin (MA5-11690) (Invitrogen)
Actin (A2066) (Sigma Aldrich)
GAPDH (0411, sc-47724) (Santa Cruz Biotechnology)
Phospho-p38 (Thr180, ab195049) (Abcam)
p38 (ab31828) (Abcam)
PSMB5 (ab3330) (Abcam)
PSMB8 (Proteasome 20S LMP7, ab3329) (Abcam)
PSMB9 (Proteasome 20S LMP2[EPR13785] ab184172) (Abcam)
Collagen I (ab6308) (Abcam)
TRAF6 (D-10) (sc-8409) (Santa Cruz Biotechnology)
IKKi (A-11) (sc-376114) (Santa Cruz Biotechnology)

NF-kB p65 (A-12) (sc-514451) (Santa Cruz Biotechnology)
TGFβ (E-AB-33090) (Elabscience)
TNFα (E-AB-40015 (Elabscience)
p62 (P0067) (Sigma Aldrich)
OPN (R&D)
Ghrelin (PA1-1070) (Invitrogen)
GHS-R (PA5-28752) Invitrogen)
LC3B (L7543) (Sigma Aldrich)
IL-10 (sc-1783) (Santa Cruz Biotechnology)
AKT (ab179463) (Abcam)
ATG7 (126M4822V) (Sigma Aldrich)
Cytokeratin 14-16 (PA5-36061) (Invitrogen)
STAT1 (ab47425) (Abcam)
Phospho-STAT1 (ab10946) (Abcam)
STAT3 (ab68153) (Abcam)
Phospho-STAT3 (ab76315) (Abcam)
AIRE (14-5934-82, 5H12) (eBioscience)
SIRT-1 (2192247) (Millipore)
CK5 (Abcam)
CK8 (Abcam)
collagen X (Abcam)
AIRE (ThermoFisher)
dystrophin (NCL-DYS-1 and NCL-DYS-2, Novocastra).

Validation

Each antibody was titrated and validated accordingly to the manufacturer instructions. All antibodies are commercially available and validated by each company for application and species uses. Further information can be found at vendors websites.

Animals and other organisms

Policy information about studies involving animals; ARRIVE guidelines recommended for reporting animal research

Laboratory animals

C57Bl (8 weeks-old and 3months-old), mdx (8 weeks-old and 3months-old) and BALB/c nude (8 weeks-old) mice were provided by Charles River and housed in a controlled ambient environment (12h light/dark cycle) at a temperature between 21°C/23°C. All the mice were males except for 4 C57Bl and 4 mdx females that were used for mating. The mice had free access to clean water and food.

Wild animals

No wild animals were used in the study

Field-collected samples

No field collected samples were used in the study

Ethics oversight

Procedures involving living animals were conformed to Italian law (D.L.vo 116/92 and approved by local ethics committees. This work was authorized by the Ministry of Health and Local University of Milan Committee, authorization number 859/2017-PR (5247B.35, 10/07/2017 and additional integration).

Note that full information on the approval of the study protocol must also be provided in the manuscript.

Flow Cytometry

Plots

Confirm that:

- [x] The axis labels state the marker and fluorochrome used (e.g. CD4-FITC).
- [x] The axis scales are clearly visible. Include numbers along axes only for bottom left plot of group (a 'group' is an analysis of identical markers).
- [x] All plots are contour plots with outliers or pseudocolor plots.
- [x] A numerical value for number of cells or percentage (with statistics) is provided.

Methodology

Sample preparation

Peripheral blood (100 µL) was collected from the mouse tail veins. Red blood cells were lysed with ACK solution (NH4Cl 150 mM, KHCO3 10mM and Na2EDTA 0,1 mM) to allow cytofluorimetric studies. For five-colours flow cytometry 105 cells were resuspended in phosphate buffered saline (PBS)
For the examination of murine thymus cellularity, thymi were depleted from fat and connective tissue, transferred to 6-well plate containing Liberase (Invitrogen) solution and incubated at 37 °C for 20 min. Then, they were dissociated as described in details in "Xing, Y. & Hogquist, K. A. Isolation, identification, and purification of murine thymic epithelial cells. Journal of

visualized experiments : JoVE, e51780, doi:10.3791/51780 (2014)" as indicated in the Matherial and Method Section
For the isolation of cTEC and mTEC, cells isolated from murine thymus were immediately enriched by thymocyte depletion - since thymus-derived cells are mainly composed of over 95% thymocytes. Accordingly, these cells were incubated with anti-CD45 antibody at a final concentration of 2.5 µg/ml: thymocytes depleted TECs were then resuspended in FACS sorting buffer and centrifuged.
For sorting, cells from peripheral blood and muscles were isolated with A-FACS Aria machine (BD Bioscience, New Jersey). For isolation of lymphocytes from muscles, TA, gastrocnemius and quadriceps muscles were excised and extensively washed in PBS to removed blood contaminants as described in details in "Burzyn, D. et al. A special population of regulatory T cells potentiates muscle repair. Cell 155, 1282-1295, doi:10.1016/j.cell.2013.10.054 (2013)". Muscles were cut in small pieces, digested for 1 hour with Liberase 0.2mg/ml (Invitrogen) and filtered with 70 µm mesh filters. Undigested tissues were mashed with a plunger through the filters and washed with DMEM additioned with serum. Histopaque (Sigma Aldrich) gradient was perfomed to separate lymphocyte fraction and centrifuges for 25 min. The T cells containing interphase was aspirated carefully, washed in PBS and stained for sorting by flow cytometry

Instrument

Data were acquired with the Cytomics FC500 (Beckman-Coulter) machine

Software

Data were analyzed with CXP 2.1 software.

Cell population abundance

For cell sorting of cTEC and mTEC from C57Bl (n=8 mice) and MDX (n=8 mice) mice, n=4 thymi/ animal group were dissociated and pooled for cell isolation, for n=2 groups/ mouse type. We isolated a total of 7.15 x 10^5 mTEC and 1.13 x 10^6 cTEC from C57Bl and 7.26 x10^5 mTEC and 1.24 x 10^6 cTEC from MDX mice. Purity of cell sorted population ranged between 99.8-100% for mTEC and 99.6-99.9% for cTEC in C57Bl mice. For mdx mice ,we determined a purity between 96.1-96.5% for mTEC and 96.2- 96.8% for cTEC. Purity was tested by the means of FACS analysis.

For intra-arterial and intra-tail vein injections, we isolated a total of 7X10^6 CD3+CD4+ cells and 5X10^6 CD3+CD8+ cells from blood samples of mdx mice (100ul/mouse), with purity ranging between 97.6-98.9% and 97-98.4%, respectively. Purity of cell sorted populations were tested by FACS analysis.

Gating strategy

Gating strategy is reported in Figures 2,3,5 and 9

☐ Tick this box to confirm that a figure exemplifying the gating strategy is provided in the Supplementary Information.
